# Emerging tools to advance neuroethology in butterflies and moths

**DOI:** 10.1007/s00359-025-01785-y

**Published:** 2025-12-10

**Authors:** Francesco Cicconardi, Max S. Farnworth, Robin Grob, Donya N. Shodja, Caroline N. Bacquet, Basil el Jundi, Arnaud Martin, Stephen H. Montgomery

**Affiliations:** 1 School of Biological Sciences, University of Bristol, Bristol, UK; 2 Department of Biology, Norwegian University of Science and Technology, Trondheim, Norway; 3 Department of Biological Sciences, The George Washington University, Washington, DC 20052 USA; 4 Universidad Regional Amazónica Ikiam, Km 8 vía a Muyuna, Tena 150101, Ecuador; 5 Institute of Biology and Environmental Sciences, Carl von Ossietzky University of Oldenburg, Oldenburg, Germany

**Keywords:** Epigenetics, Single-cell transcriptomics, Transgenics, Neuroecology, Neurophysiology

## Abstract

Butterflies and moths have played historically important roles in developing our understanding of both ecology and evolutionary biology, and neuroethology. In both contexts, the diversity of behavioral strategies and specializations displayed by different Lepidoptera make them informative case studies. However, as in neuroscience more broadly, lepidopteran neuroethology has tended to focus on intricate functional studies within a small number of the most tractable species. In contrast, ecologists and evolutionary biologists have often taken a broader view, using phylogenetic and comparative approaches to extract general patterns of diversification, and to exploit the diversity of butterflies and moths to understand general evolutionary processes. Uniting these approaches and traditions has been restricted, largely due to technical challenges of working with unestablished study systems and a lack of resources beyond basic tools. Now, however, the prospects for broader comparative studies of the neural basis of behavior within a phylogenetic and/or ecological framework are increasingly positive. This is in large part due to the emergence of new molecular sequencing approaches and associated tools. These allow for the survey of cell types, the spatial location of their soma, development of new cell-type markers for targeted analyses, and quantification of the dynamic regulation of gene expression at a tissue or cell specific level. Results of these molecular methods can be combined with technical developments in free flying behavioral experiments in tethered animals that permit neural recordings of natural behavior, and functional genetics tools that can allow for more precise manipulation of these behaviors or the neural structures that support them. Here, we review these new approaches, their potential application, and discuss how we can use them to advance the development of new, integrative systems for studying the neural basis of behavior in butterflies and moths.

**Supplementary Information:**

The online version contains supplementary material available at 10.1007/s00359-025-01785-y.

## Introduction

Understanding how nervous systems produce behavior is the central aim of neuroethology, and the huge diversity of animal behavior provides almost endless inspiration for this endeavor. Indeed, neuroethology has a long tradition of leveraging a range of species with particular specializations, each suited to asking questions about specific behaviors or neural processes (Carlson 2012; Yartsev 2017). However, in broader terms, 20th century neurobiology has increasingly focused on a few “model organisms”. This strategy has been a success, leading to a range of tools that enable us to understand and manipulate behavior at the circuit level in a few select species (Bellen et al. 2010; Anderson and Ingham 2003). Work on neurobiology in these model species also underlines the links that can be drawn across animal systems. For example, genes regulating neurogenesis (Robinson et al. 2020; El-Danaf et al. 2023) and cell identity (Bier 2005; Holguera and Desplan 2018) illustrate conservation of function between *Drosophila* and vertebrates, validating insect models for fundamental neurobiological questions (e.g. Bilen and Bonini 2005; McGurk et al. 2015). However, the range of trait variation reflected by any single species is naturally limited. Therefore, not all questions can be addressed in any single taxa, and the dominance of a few species limits our power to generalize functional inferences, in at least some contexts (Carlson 2012; Yartsev 2017; Laurent 2020; Hale 2019; Mathuru et al. 2020; Jourjine and Hoekstra 2021). Broadening our range of model species is the core path to addressing these concerns and will allow us to gain a more complete insight into the function of neural circuitry.

At the same time, to understand how brains produce each species’ behavioral repertoire, an appreciation of the environment in which those brains evolved and operate is crucial (Carlson 2012; Mathuru et al. 2020; Jourjine and Hoekstra 2021). Hence, leveraging species with well understood, variable ecologies has clear benefits. Until recently, the lack of advanced tools made establishing new study systems intractable. However, increasingly, new techniques make developing novel, complementary models a realistic prospect. In doing so, a critical first step is identifying axes of neural variation across tractable species that have high potential to offer novel insights into fundamental biological processes that regulate the development of complex systems.

**Fig. 1:**
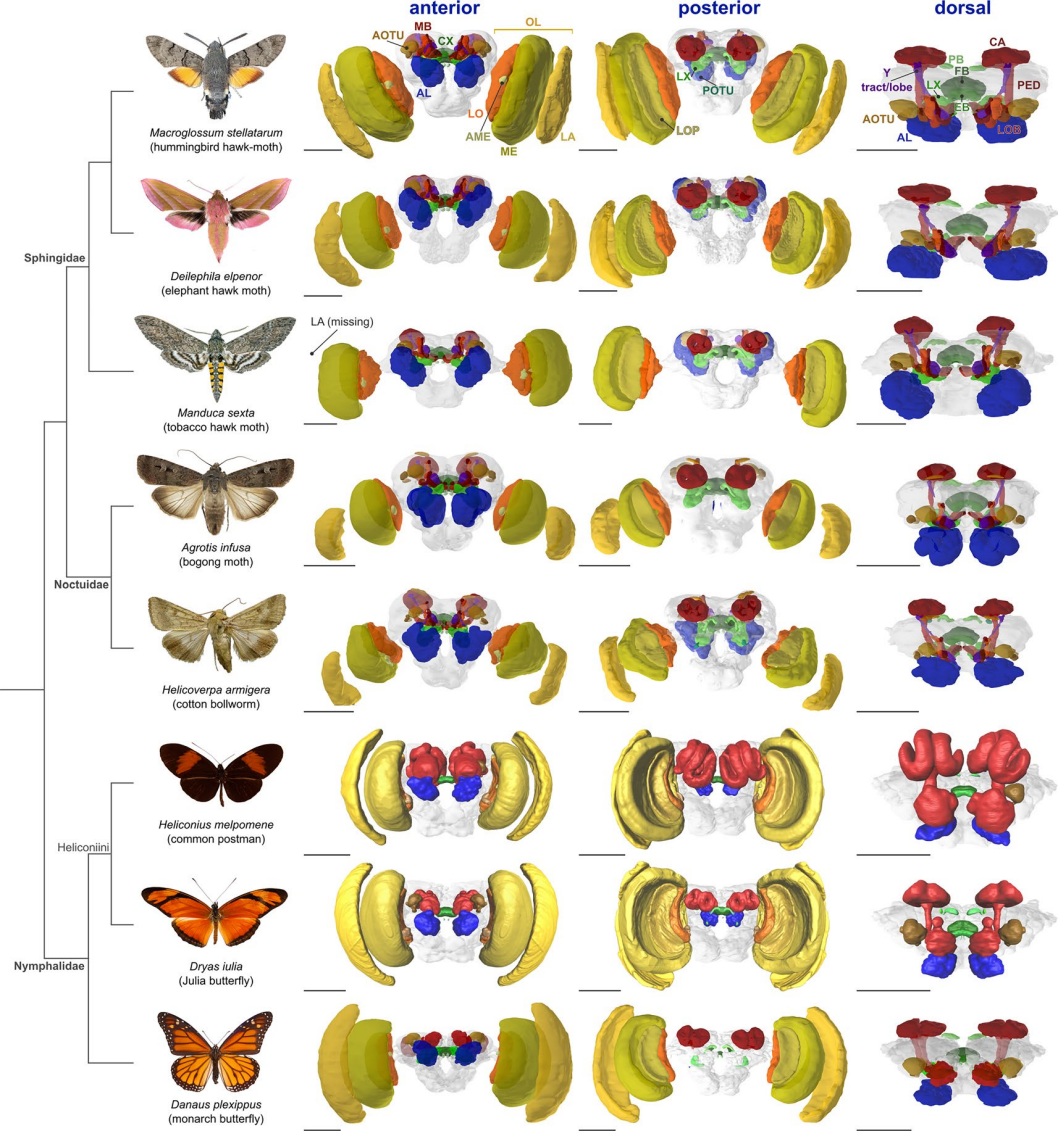
Diversity of Lepidoptera brain anatomy. Lepidoptera share a largely common set of neuropils, synapse dense regions with specific functions. These include hemispherically symmetrical sensory neuropils predominantly in the optic lobes (processing visual information) and antennal lobes (processing chemosensory information), and integrative centres such as the mushroom bodies (the site of insect learning and memory) and the central complex (the site of spatial orientation and locomotor control). For a full review of lepidopteran brain anatomy see Adden et al. (in review). However, while generally conserved in presence/absense, the size of these neuropils can vary extensively across species, as represented in this figure. Shown are three views of the brain: anterior, posterior, and dorsal (relative to neuraxis), with prominent neuropils. Anatomical data for all except for Heliconiini species were sourced through https://insectbraindb.org/ (Heinze et al. 2021). Heliconiini data was generated by the authors. In Heliconiini (here, *Heliconius melpomene* and *Dryas iulia*), undefined neuropils of the central brain surrounding the coloured neuropils, which are depicted in all other species in grey, were not included. In *Dryas iulia*, hemispheres were mirrored. Scale bar is 500 μm. Abbreviations: AOTU anterior optic tubercle, MB mushroom bodies, CX central complex, AL antennal lobe, LO lobula, AME accessory medulla, ME medulla, LA lamina, OL optic lobe, LOP lobula plate, LX lateral complex, POTU posterior optic tubercle. FB fan-shaped body, PB protocerebral bridge, EB ellipsoid body, LOB mushroom body lobes, PED peduncle, CA calyx. The phylogenetic tree was generated using phyloT v2 at https://phylot.biobyte.de/. Image credit of Lepidoptera: *Macroglossum stellatarum* - Didier Descouens; *Deilephila elpenor* - Didier Descouens; *Manduca sexta* - Didier Descouens; *Helicoverpa armigera* - Dumi (Author), CC BY-SA 3.0, *Agrotis infusa* - Birgit E. Rhode, CC BY 4.0; *Danaus plexippus* - Didier Descouens; *Dryas iulia* - Didier Descouens; *Heliconius melpomene* - Notafly (Author), CC BY-SA 3.0. On all images, background was removed. All images were sourced through https://commons.wikimedia.rg/ and were published under a CC BY-SA 4.0 license if not otherwise specified

In this context, Lepidoptera are exceptionally well placed to play a significant role in the next wave of neuroethological model systems. A major reason for this is a long, parallel history of Lepidoptera as study systems in both neuroethology, and ecology and evolution. In a neuroethological context, Lepidoptera have made major contributions to our understanding of specialisations in sensory perception in both olfactory (e.g. Hansson et al. 1992; Berg et al. 1995) and visual contexts (e.g. Swihart 1964; Swihart 1972; Steiner et al. 1987), while understanding specific behavioral traits, in particular long-distance migration (Beetz et al. 2022, 2023; Dreyer et al. 2025) and navigation (Grob et al. 2025), have become major case studies in goal-oriented behavior. Similarly, in an ecological and evolutionary context butterflies and moths have provided productive case studies in adaptive divergence across habitat types (e.g. Montgomery et al. 2021; Wainwright et al. 2024) and diel activity pattern or sensory conditions (e.g. Kawahara et al. 2018; Sondhi et al. 2021). They have illustrated the importance of behavior during speciation in the context of mating (e.g. Jiggins 2008; Merrill et al. 2011) and host plant preferences (e.g. Janz and Nylin 2008; Fordyce 2010), and substantial progress has been made in understanding the molecular or sensory basis of these behavioral decisions (e.g. Rossi et al. 2024; VanKuren et al. 2025). Importantly, work in these systems also has a long tradition in phylogenetics, meaning the relationships within and between most lineages of Lepidoptera (Mitter et al. 2017; Kawahara et al. 2019, 2023), and in particular, well studied radiations of butterflies (e.g. Kozak et al. 2015; Lisa De-Silva et al. 2017; Cicconardi et al. 2023; Condamine et al. 2023), are well understood. This provides an essential framework for comparative studies that, in conjunction with ecological data, help to identify clades that present striking behavioral diversity or innovations that may be amenable to a neuroethological approach. Indeed, increasingly, there is clear recognition that the diversity of Lepidoptera is reflected, to some extent at least, in the presence of divergent specialisations in sensory and neural systems (e.g. Montgomery et al. 2016, Montgomery and Merrill 2017; Stöckl et al. 2016; de Vries et al. 2017; Couto et al. 2020; Fig. 1). Finally, across neuroethology, ecology and evolution, work in Lepidoptera has often been at the forefront of new methodologies, from now classic experimental systems such as electroantennograms, first developed in moths (Topazzini et al. 1990; Raguso et al. 1996), to pioneering work to assemble some of the first insect genomes (Mita et al. 2004; Zhan et al. 2011; Dasmahapatra et al. 2012), and early adoption of gene editing methods (e.g. Tamura et al. 2000; Uchino et al. 2007).

Lepidoptera therefore provide many opportunities to advance our understanding of the neural basis of behavior, while the challenge of developing resources for new study systems is now much more feasible. Nevertheless, it is useful to identify the core ‘tool kit’ needed to establish productive case studies (Jourjine and Hoekstra 2021; Mathews and Vosshall 2020). Ideally, this toolkit will often include: (i) brain atlases to identify circuits of interest and neuroanatomical variation; (ii) an understanding of the dynamics of gene regulation in environmentally sensitive circuits, to link molecular and neural activity; (iii) an ability to record neural activity in ecologically relevant settings; and (iv) genomic resources to identify cell markers, and transgenic methods that enable us to observe and manipulate specific cell types and behaviors. Here, we discuss current and developing methodologies in lepidopteran neuroethology, and how they can be combined to allow greater exploitation of the behavioral and neural diversity of butterflies and moths.

## Molecular tools to study neural diversity in Lepidoptera

Neurons in the insect brain can be grouped into different types, which differ in their morphology connectivity pattern, molecular identity and physiology. Thus, each neuron type is tuned to respond to specific internal and external cues, within the functional context of a wider neural circuit (Arendt et al. 2016; Zeng 2022). Identifying neural cell types and circuits, revealing what cues they encode to ultimately understand their functional diversity and how they vary across individuals, sexes, or species is a central goal in neuroethology (Bates et al. 2019; Zeng 2022). Here, we introduce standard techniques to define and categorize neuron types, and contrast their advantages and limitations with emerging sequence-based methods, before discussing the opportunities and challenges ahead of developing detailed neural atlases in Lepidoptera.

### A molecular approach to defining neuron types

Traditionally, neural cell types have been characterized in lepidopterans based on their morphology and physiology using intracellular recordings combined with tracer injections, in which dyes are injected into neurons which subsequently fill the cell body, enabling cell morphology and spatial arrangement to be visualised precisely (e.g. Kinoshita et al. 2015; Nguyen et al. 2021). However, this method does not always allow us to unambiguously identify homologous neuron types within and across species because homologous neurons may have divergent morphologies, while having conserved molecular profiles (Arendt et al. 2019). Interindividual variation in the expression of specific peptides or transmitters in homologous neurons, changes in morphology and physiology across an animal’s lifespan, and dynamic neural coding makes the characterization of cell types even more challenging (Zeng 2022). The recent emergence of single-cell or single-nuclei sequencing technologies provides an alternative way to identify neural cell types, and is set to revolutionize how we approach the cell biology of neural systems. These methods form cell clusters enabling cell types to be identified based on the genes each cell expresses (the ‘transcriptome’, profiled by reading the transcribed mRNA sequences) and their expression levels (i.e. how much mRNA for each locus is present in a cell). A key advantage is that a sequencing approach is agnostic, scalable, and comprehensive (Nawy 2014), essential criteria when trying to make a global assessment of neuron types in non-model species (Zeng 2022). Supplemented by single-cell epigenomic approaches (such as scATAC-seq for determining chromatin accessibility), many more genetic dimensions can be captured to help classify neurons. It is further expected that many cell-type determining genes are highly conserved across insect clades such as Lepidoptera (Arendt et al. 2016, 2019; Hobert and Kratsios 2019), which provides a robust basis for comparing homologous cell types between different lepidopteran species.

### Single-cell sequencing to catalogue neuron types

A wide range of experimental techniques have been developed for both single-cell (scRNA-seq) and single-nuclei (snRNA-seq) RNA sequencing. However, performing scRNA-seq in neural tissue is not trivial as it requires isolating intact neurons, which in insects exhibit a complex morphology of unipolarity where the nucleus is far removed from pre- and postsynaptic sites. Thus, snRNA-seq has been the method of choice in many insects in the past, especially because early studies suggested that the scRNA-seq and snRNA-seq gene expression patterns are highly similiar (e.g. Ding et al. 2020). Beyond this distinction between having cells or nuclei as starting material, most approaches developed to date are applicable to both forms of starting material, and we therefore use scRNA-seq as a catch-all term. We briefly describe the major steps in the approach, and discuss their application in Lepidoptera.

**Fig. 2:**
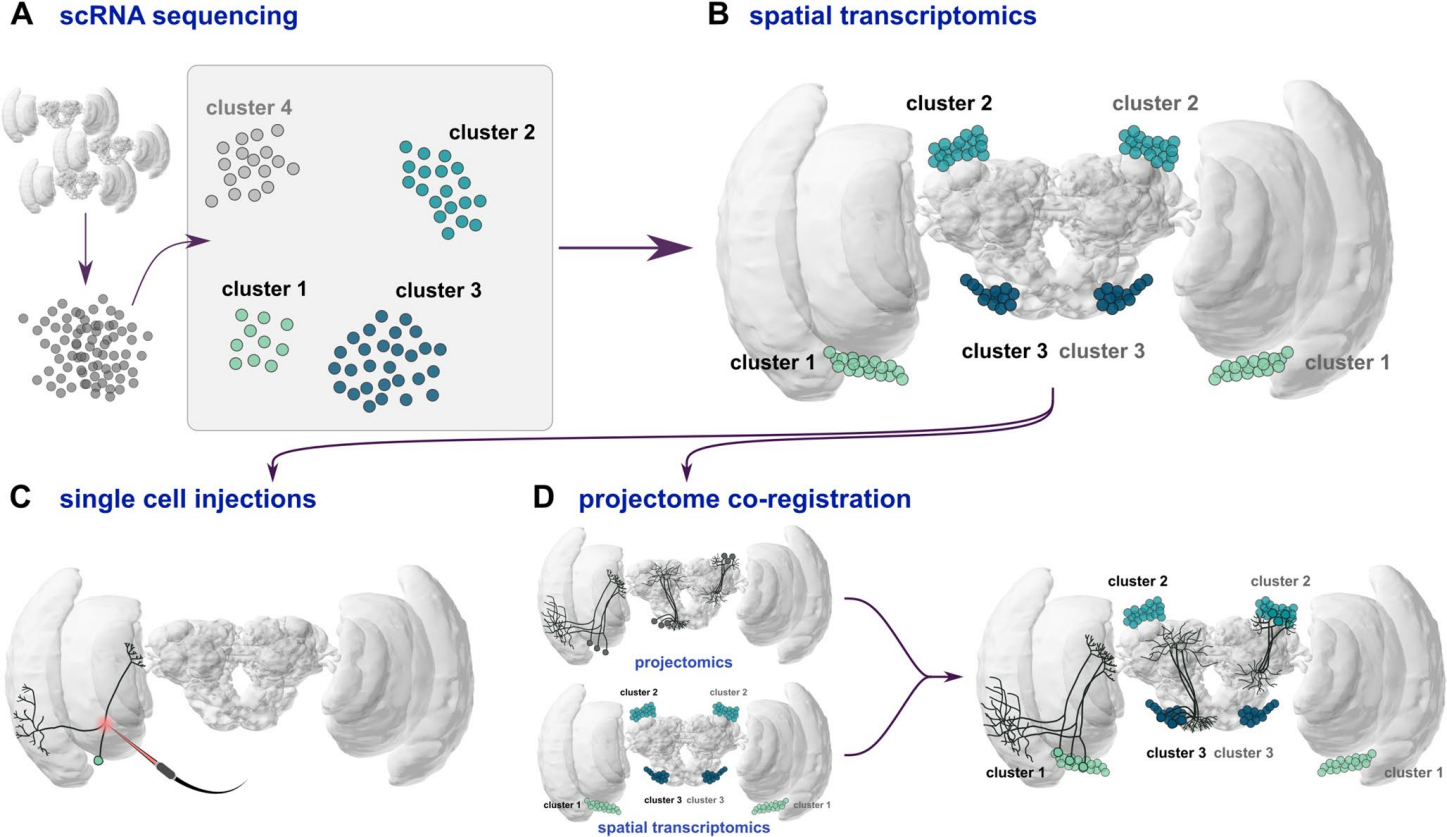
Schematic workflow for the Integration of molecular cell type information and anatomical information of soma and cell projections. **A.** Schematic depiction of the isolation of single nuclei from Lepidoptera brains, and subsequent identification of four cell types. **B.** spatial transcriptomics then identifies the relative location of these cell types in the Lepidoptera brain. **C.** The gathered information about neuron types can then be corroborated through morphological means, firstly using single-cell injections (electrode icon through bioicons.com). **D**. Secondly, projectomics approaches can be performed which then can be co-registered with spatial transcriptomics information to generate a full-scale morphology/genetics combined atlas. Brain shape is from the *Danaus plexippus* brain available at https://insectbraindb.org/ (Heinze et al. 2021)

scRNA-seq methods can generally be classified based on the strategy that they implement to separate the molecular signal from each cell (Table S1). A key step in all methods is the isolation of individual cells (or nuclei) to permit the genes transcribed within to be assayed independently of others. The method of isolation varies, and among the first implemented methods relied on manual sorting of individual cells into multi-well plates (Picelli et al. 2014; Wei and Lee 2025) or tiny droplets (Danielski 2022; Kim and Marignani 2022). This droplet approach was commercialized by 10X Genomics Chromium, and is currently one of the most common methods. Droplet approaches are known for their scalability and efficiency in processing large numbers of cells, but they generally have low capture efficiency, require special equipment, and can have high ‘multiplet’ rates; multiple cells encapsulated in a single droplet, which can significantly confound downstream analyses. More recently, combinatorial indexing methods (or split-pool barcoding) have been developed, promising to overcome some of these limitations. These indexing methods add unique ‘barcodes’ of sequence to each RNA molecule without the need for physical isolation of cells (e.g. Kuijpers et al. 2024; Li et al. 2023). Unique barcodes can be provided to many thousands to millions of cells, dramatically increasing the scale of cell sampling for a given cost. This ultra-high throughput capability allows the simultaneous processing of vast numbers of cells, making them ideal for large-scale studies, or for reducing batch effects by pooling samples, with their group identity (e.g. species/sex) preserved in the barcodes. With the resulting samples, the transcriptomic profile of each individual cell can be sequenced and used to hierarchically cluster all cells within a sample, linking those with similar profiles and grouping them into broader classifications (2022) (Fig. 2A). These datasets are the key basis for defining molecular cell types in an unbiased and generalizable way. These data in themselves can also reveal surprising biology, for example, scRNA-seq has been used to demonstrate co-expression of olfactory receptors within single sensory neurons in mosquitos (Herre et al. 2022), contrary to long-held views that a 1:1 relationship always exists between olfactory neurons and olfactory receptor expression.

For many species, a remaining challenge is to assign identified cell clusters names and putative functions. This has been most readily done in model species where cell-specific markers are already available (e.g. Davie et al. 2018; Brunet Avalos et al. 2019), but the extension of cell markers across species can be problematic due to technical artefacts or biological diversity, particularly for more precise cell classifications. Nevertheless, scRNA-seq data are directly useful in generating catalogues of cell types, which then permits comparisons of cell composition (i.e. the presence and relative abundance of different cell types in a tissue) across groups, such as species or sexes, at a level of detail that cannot currently be achieved with traditional staining and imaging methods. For example, in *Drosophila melanogaster*, scRNA-seq has been used to characterise sex-specific sensory organs in the foreleg, partitioning out chemosensory and mechanosensory structures (Hopkins et al. 2023), to provide evidence that sexual dimorphism in neural function is not due to sex-specific cells, but rather sex-specific gene regulation operating within common cell determination programs (Palmateer et al. 2023), and to precisely map the molecular diversity of cell types in the *D. melanogaster* nerve chord and connect these cell types to the nerve chord connectome (Cachero et al. 2025).

To date, very few studies have used scRNA-seq experiments to study the brain or sensory systems of moths and butterflies. Instead, one of the first applications of this technique have been in understanding the midgut, to study the dietary physiology or immune response of agricultural pests (*Spodoptera fruigiperda/Plutella xylostella*) (Arya et al. 2024; Xia et al. 2024; Chen et al. 2025; Sun et al. 2025), or the silk gland in *Bombyx mori* (Ma et al. 2014). A second major application has been to study the evo-devo of wing patterns (Prakash et al. 2024; Loh et al. 2025). Here, scRNA-seq has been central to establishing the developmental origins of scale cells (Loh et al. 2025), and for understanding how cell fate is determined by gene expression patterning (Loh et al. 2025; Prakash et al. 2024), questions that have clear analogues in the development of sensory and neural traits in Lepidoptera. To date, we know of only two studies focused explicitly on lepidopteran brains, both on *Bombyx mori*. Liu et al. (2024) sequenced ~ 50,000 cells from larval and adult *B. mori* to catalogue neural cell types and explore the cellular composition of a lepidopteran brain, demonstrating expected shifts in cell composition between life stages in comparison to other insects. Feng et al. (2024) focused instead on the change in gene expression in brain cells following infection by *B.mori*-nucleopolyhedrovirus (BmNPV), revealing an important immune role for lysozyme expression within hemocytes.

The gene expression profiles that define many cell types are also expected to be well conserved across species, which can therefore allow for the integration of profiles across different species, sexes, or groups based on behavioral phenotypes/states (Arendt et al. 2016, 2019; Hobert and Kratsios 2019). Recent work among closely related *Drosophila* species, for example, has revealed divergence in cell composition within *D. sechellia* brains, an ecological specialist, with putative roles for divergence in the abundance and gene expression profiles of specific glial cell sub-types in the genetic and physiological adaptation to their novel food source (Lee et al. 2025). In Lepidoptera, integration of molecular cell types across species would allow for comparative analyses of homologous cell types across species with neural traits or ecologies of interest, including direct quantification of a cell type diversity and abundance. For example, the well characterized diversity of butterfly color vision systems (Arikawa 2017 ) and associated circuitry (Matsushita et al. 2022), the extreme and repeated evolution of sexually dimorphic lepidopteran olfactory systems (Rospars and Hildebrand 2000; Morris et al. 2021), neural specializations in integrative centres (e.g. Couto et al. 2023), and the frequent occurrence of seasonal polyphenism (Nylin 1994; Halali et al. 2024), are all biological phenomena that are well explored in Lepidoptera, where scRNA-seq could provide new insights into the cellular or molecular basis of behavioral traits by revealing changes in cell type abundance, changes in gene expression within specific cell types or the innovation of new cell types.

### Integration of anatomical information with genetically defined cell types

Catalogues of molecular cell types are a major step towards a precise atlas of neural pathways, which is critical to develop a system for broad use as a neuroethological model. Cells are not isolated entities and reside in complex microenvironments and are deeply influenced by neighboring cells to collectively shape the functional properties of tissues (Palla et al. 2022). scRNA-seq does not capture the context of a cell’s microenvironment, so determining the location of a cell type’s soma is a critical next step for understanding tissue architecture (Crosetto et al. 2015; Asp et al. 2020), particularly in cases where assigning identities to cell clusters based on uncharacterized genes is challenging, such as in poorly studied non-model species. This is because inferring the identity of cell clusters based on the expression of genes known to accurately denote cell-types in other species may be unreliable, due to a lack of conservation in the specificity of expression in those genes across species. However, new species-specific cell markers, genes whose expression defines molecular cell type, can be developed for downstream analyses to confirm that these cells exhibit the predicted spatial expression patterns of their assigned identities. For example, recent developments in in situ Hybridisation Chain Reaction (HCR) across multiple non-model organisms, offer a scalable alternative to immunohistochemical staining to link specific cell types to their spatial location in neural or sensory systems (Choi et al. 2018; Tsuneoka and Funato 2020). Multiplex methods, where fluorescent tags that emit different wavelengths are used to visualize multiple target genes simultaneously, have been used in butterflies to study gene patterning in developing wing discs (Banerjee et al. 2024) and larval brains (Banerjee et al. 2025).

However, more global comparisons will soon benefit from retention of spatial information in transcriptomic data. Sequence-based spatial transcriptomics (sST) enables comprehensive transcriptomic profiling of cells in a tissue of interest while preserving spatial information (Hickey et al. 2023; Greenwald et al. 2024) (Fig. 2B). This field is still in its infancy, with few studies in insects (Ma et al. 2024; Janssens et al. 2025). Nevertheless, methods are developing rapidly (Table S2). Unlike imaging-based techniques such as FISH or in situ sequencing, which require the design of probes for predefined genes, spatial transcriptomics enables unbiased, whole-transcriptome profiling, making it especially valuable when studying poorly characterized tissues (Gulati et al. 2025). The general principle of spatial transcriptomics is to capture mRNA from tissue sections while maintaining spatial information, prior to high-throughput sequencing. The preservation of spatial information can be achieved through a variety of methods, for example using arrays of spatial barcodes to encode a specific location within the sequence data, or beads that capture RNA molecules for in situ sequencing (Table S2). Currently, spatial resolution is limited, which is particularly problematic for small, densely packed tissues like lepidopteran brains, but available technologies are improving rapidly. Nevertheless, there is clear promise in the dual use of scRNA-seq and spatial transcriptomics to drive the creation of brain atlases at a level of precision previously limited to model organisms. This approach has recently been applied to *Drosophila* brains, revealing the spatial location of large cell clusters in the brain (Janssens et al. 2025), and in *Bombyx mori* where it was used to profile the spatial and temporal regulation of gene expression in the silk gland (Ma et al. 2014). In the context of lepidopteran neuroethology, this approach would be sufficient to provide a spatial reference of major cell types, for the first time, which can be used to direct a range of studies, including neurophysiological assays of neural activity, and transgenic experiments to knock out, label or modulate specific cell types.

A particularly exciting prospect for understanding the diversity of neural cells and circuits is the integration of catalogues of molecular cell types, the spatial location of their cell bodies through spatial transcriptomics, and projectomic or connectomic maps of neural connectivity (Fig. 2). This combination of molecular and precise anatomical information can be achieved for specific cells by integrating cell type markers with traditional single-cell injections (Fig. 2C) which label isolated, but targeted neurons, in a highly specific way. These targeted neurons, and the location of the cell bodies obtained through spatial transcriptomics, can then be warped into a 3D brain atlas. This enables precise identification of each neuron and its relative position within the brain. Comprehensive brain atlases are already available for several lepidopteran species (el Jundi et al. 2009; Adden et al., 2020; de Vries et al. 2017) and are publicly accessible (Heinze et al. 2021). These resources can allow researchers to map and visualize cell clusters, defined by scRNAseq in combination with HCR or spatial transcriptomics, in relation to well-characterized brain structures and neurons, facilitating predictions about their roles in various behaviors. While we are still at an infancy to combine these distinct genetic and morphological methods and anatomical assessments based on single cell injections are highly specific to select neurons, there are additional prospects to develop such neural connectivity maps at a global scale. Currently, connectomics are highly taxonomically limited to a small handful of invertebrates (Cook et al. 2019; Scheffer et al. 2020; Schlegel et al. 2024). However, developing methods which apply X-ray (Hwu et al. 2017; Laugros et al. 2025) or light microscopy (Tavakoli et al. 2025) as an imaging platform, rather than electron-microscopy, may rapidly change the landscape of this field by being quicker and more broadly accessible. Co-registration of spatial transcriptomic atlases with these novel anatomical maps should allow cell-type specific pathways to be reconstructed, potentially alongside their inter-cellular connections (Fig. 2D). Registration methods and reconstruction of projectomes in this budding field may be achieved by deep-learning-based approaches (Tavakoli et al. 2025). In addition, the generation of projectomes and connectomes may be aided by cleared tissue, without any reflective obstructions in a way of precise imaging (Susaki et al. 2014). These novel approaches would facilitate a new wave of advancement in comparative connectomics, building on established behavioral and functional models of the *Drosophila* connectome (Schlegel et al. 2024; Scheffer et al. 2020; Lin et al. 2024).

In summary, cataloguing the diversity and location of cells within neural tissue is of fundamental importance, unlocking the door to a range of neuroethological questions and experiments. Integrating molecular data on cell types with anatomical data provides a particularly powerful way of understanding brain architecture (Bates et al. 2019; Zeng 2022; Schlegel et al. 2024). Achieving these links between cells clustered by gene expression, and cells defined by morphology and function remains a major challenge even in model organisms at the forefront of these developments (Bates et al. 2019; Schlegel et al. 2024; Zeng 2022; Cachero et al. 2025). However, for the first time, it is a viable objective to work towards this goal in Lepidoptera. Achieving this goal will rapidly build on the anatomical and behavioral insights already achieved in Lepidoptera, and will allow us to integrate neuroethological approaches with the strong traditions of phylogenetic, behavioral and ecological research in Lepidoptera.

## Dynamic gene regulation of neural cells

While parts of a cell’s identity are static, neural cell plasticity is central to behavioral flexibility (Zovkic et al. 2013; Gegner et al. 2021), and there is a great deal of interest in behavioral plasticity in Lepidoptera, either in the context of polyphenism (e.g. Nylin 1994; Halali et al. 2024), or behavioral processes like learning and memory (e.g. Van Dijk et al. 2017; Snell-Rood 2013; Connahs et al. 2022). In the past, the activity of neurons has been monitored through electrophysiological recordings in butterflies and moths (see Section “Advances in neurophysiological recordings in free moving Lepidoptera”). However, novel molecular techniques are now available that can be applied to gain insights into the physiology of neurons in lepidopterans. Environmental stimuli, metabolic states and developmental signals all trigger changes in gene expression, chromatin accessibility, and epigenetic modifications, allowing neurons to integrate internal and external information over time. Changes in epigenetic markers, such as DNA methylation or histone modification, can alter the transcriptional activity of neural genes, leading to modifications in neuronal activity, often with remarkable speed and specificity. These dynamics are fundamental to animal behavior, but rarely studied in Lepidoptera (Jones et al. 2018; Velikaneye and Kozak 2025; Boman et al. 2023). In insects, although global DNA methylation levels are lower than in vertebrates, DNA methylation and histone modifications have been linked to physiological and behavioral plasticity (Maleszka 2016; Luo and Zhou 2024). More broadly, the coupling between epigenetic states, gene expression, and behavioral plasticity allows some insects to adapt their cognitive and physiological responses to changing ecological contexts. Again, recent technological developments, particularly molecular methods, provide new opportunities to pursue questions in this area. Here, we will briefly introduce established and new methods, and discuss their advantages and limitations, and the opportunities they provide in advancing neuroethology in Lepidoptera.

### Established methods of profiling methylation

To study DNA methylation at high resolution, several sequencing-based methods have been developed that have greatly expanded our ability to detect and quantify cytosine modifications at single-base resolution. Traditional approaches such as whole-genome bisulfite sequencing involve the chemical conversion of unmethylated cytosines to uracils, data which is then captured when those nucleotides are sequenced, enabling precise mapping of 5-methylcytosine (5mC) across the genome. This approach has been instrumental in uncovering how epigenetic modifications regulate insect development and behavior. For example, using bisulfite-sequencing, food stress has been shown to alter genome-wide patterns of methylation in head tissue of painted lady butterflies (*Vanessa cardui*) (Boman et al. 2023), suggesting a potential mechanism for behavioral decision making that links environmental effects of gene regulation and behavior. Indeed, in other insects, bisulfite sequencing has revealed differential DNA methylation patterns associated with task specialization, such as the transition from nursing to foraging (Foret et al. 2012), while experiments using DNA methyltransferase inhibition, which blocks the enzymes that add methyl groups to DNA, have shown that disrupting methylation patterns impairs olfactory learning and memory (Biergans et al. 2015). In the sphinx moth, *Manduca sexta*, methylation sequencing has also highlighted extensive methylation reprogramming during metamorphosis, associated with the remodeling of neural circuits that underlie adult behaviors (Gegner et al. 2021). Together, these applications illustrate how dynamic DNA methylation patterns contribute to behavioral plasticity and development in insects.

### Emerging methods of profiling methylation

The great majority of previous studies have used chemical treatments to isolate methylation signals using short-read sequencing. The advent of long-read sequencing (LRS) by Pacific Biosciences (PacBio) and Oxford Nanopore Technologies (ONT) provides a new approach that offers improved accuracy, without the need for additional protocols beyond DNA/RNA extraction. Both these sequencing technologies can directly sequence native DNA molecules, and because they can natively detect a change in modified nucleotides (DNA/RNA), no additional library preparation steps are required to enable the detection of DNA methylation. In addition, because LRS operates at a single-molecule resolution without the need for amplification, it can provide a more quantitative and accurate measurement of epigenetic modifications. New tools, designed specifically for interpreting epigenetic signals in DNA sequence data can detect specific categories of methylation profiles, which may have specific effects on transcriptional activity (Liu et al. 2021). The extended read lengths of ONT and PacBio can also enable the phasing of methylation patterns with genetic variants, enhancing the detection of allele-specific methylation. This is particularly valuable in understanding the regulatory mechanisms that underpin intra-specific behavioral variation.

Methylation profiling using LRS is a relatively new approach compared with standard techniques, and few published studies exist in Lepidoptera. However, recent applications of this approach demonstrate its strengths. LRS genome-wide DNA methylation profiles have a been generated on the fall armyworm (*Spodoptera frugiperda*), where differences in methylation of pesticide-tolerant and -susceptible strains were found, alongside evidence that a reduction in methylation density within the gene body of a 3’,5’-cyclic nucleotide phosphodiesterase gene resulted in decreased expression and increased tolerance to the pesticide (Zou et al. 2024). Work in other insects has also demonstrated roles of methylation in suppressing transposable elements (Qiu et al. 2023), and in shaping gene regulation across developmental stages and intraspecific morphs (Chavarria et al. 2025). Finally, as discussed above, new technologies have opened up transcriptomic profiling at a cellular level. Here too, advances have been made in the profiling of epigenetic features such as DNA methylation and DNA accessibility (Angermueller et al. 2016), with new methods that provide a single approach to transcriptomic and epigenetic profiling of single cells on the horizon.

In summary, behavioral variation is not just the product of static cells and circuits, but the dynamic regulation of gene expression in a context-specific manner. Understanding this process at a cellular level is therefore central to understanding the neural basis of behavioral diversity, within and between species. New long-read sequencing technologies have significantly advanced the study of DNA methylation, particularly in non-model organisms. These platforms enable direct detection of base modifications without the need for chemical treatments, offering insights into epigenetic regulation across diverse species. Because of the relative ease of these approaches compared to previous methods based on chemical treatment, it is very likely that, in the next years, we will see an expansion of these methodologies applied to different systems, including Lepidoptera, where epigenetic changes in gene regulation may well play a critical role in many behavioral polymorphisms within species, ontogenetic changes across the lifespan, or to facilitate learnt behaviors.

## Advances in neurophysiological recordings in free moving Lepidoptera

**Fig. 3:**
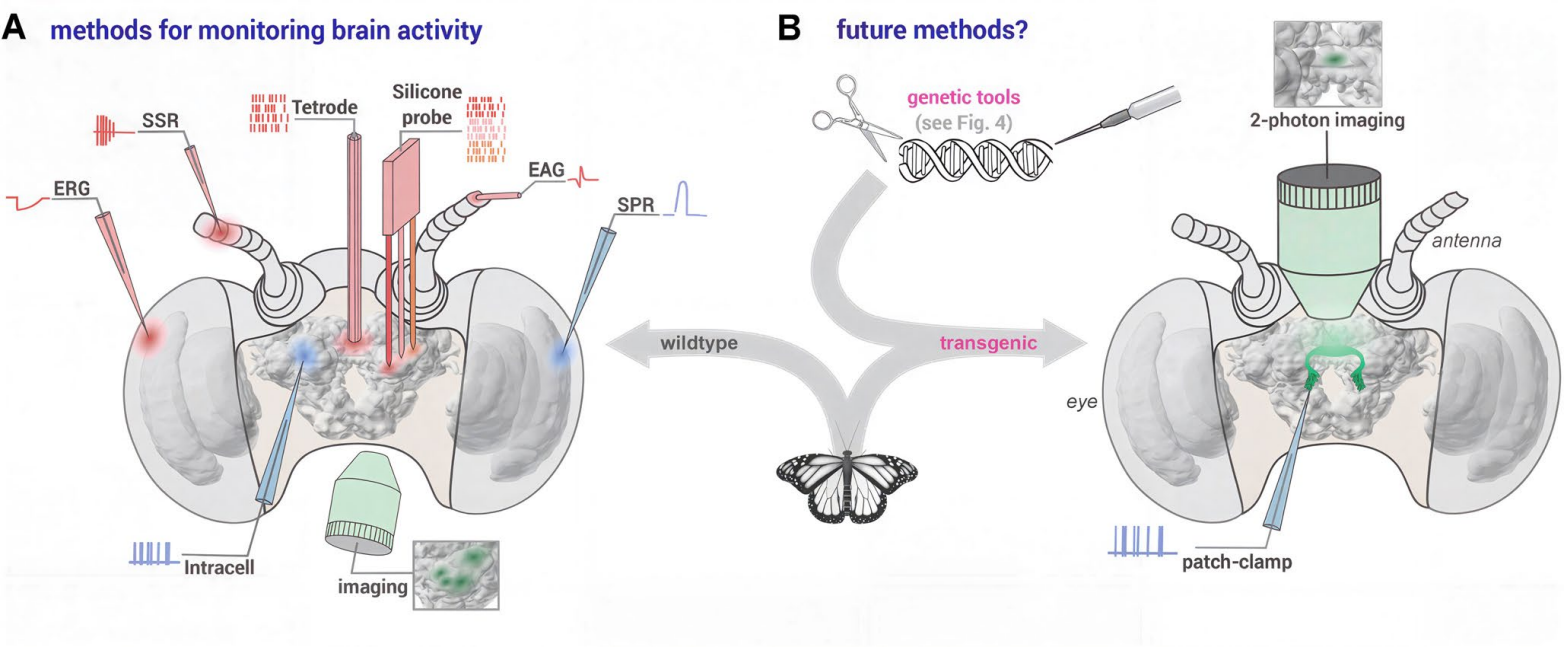
Neurophysiological methods applied to observe neural activity. (A) Anterior view of a lepidopteran head, with the head capsule opened frontally. As an example, the Monarch butterfly brain is shown (from Heinze et al. 2013). All methods applied in moths and butterflies so far are shown schematically. Methods that record the neural activity based on extracellular recordings are indicated in red, techniques for monitoring neural activity intracellularly are shown in blue, and approaches that allow imaging neural activity are shown in green. EAG: electroantennography; ERG: electroretinography; SPR: single photoreceptor recording; SSP: single sensillum recording. (B) The same as in A but with neurophysiological techniques that could be applied in the future by developing transgenic lines based on genetic tools (see Fig. 4). This would allow monitoring neural activity from identified neurons

Molecular approaches help us to determine the diversity of cell types, and how their regulatory dynamics may shape behavioral variation. However, behavior is ultimately the product of electrical communication between cells within a circuit, and as such understanding this dimension of neural activity is central to neuroethology. Due to their ecological impact, large behavioral repertoire, and ability to adapt to specific environments, several lepidopteran species have already become established model systems in neurophysiology. To gain insights into how these lepidopterans perceive their world, how their brains integrate multiple environmental cues, and how these cues are used to control diverse behaviors, a wide range of neurophysiological techniques, established over the past 50 years, have been invaluable (Fig. 3A). Here, we briefly introduce these methods, explain their advantages and limitations, and outline the technological gaps that need to be filled in the future to make the next big steps in exploring neural circuits and their role in controlling lepidopteran behavior.

### Neural recordings in static butterflies and moths

Many moths, such as the male silk moth (*Bombyx mori*), are known for their conspicuous antennae. Electroantennography (EAG), often combined with a gas chromatography (GC-EAD) (Chan et al. 2024; Fraser et al. 2003) has been applied to investigate which olfactory cues are detected by the lepidopteran antennae (Malo et al. 2004; Shiota et al. 2021). In this method, volatiles are presented to an antenna dissected from the head, and the summed response of olfactory receptor neurons, represented by a change in electric potential, can be observed through an electrode inserted into the antennal nerve. This technique permits comparisons between the antennal responses of males and females (Raguso et al. 1996), or different butterfly species (Topazzini et al. 1990). While EAG/GC-EAD recordings can be applied to qualitatively study antennal responses, single sensillum recordings (SSR) are the method of choice to quantitatively investigate the sensitivity of olfactory receptor neurons (ORNs) in the lepidopteran antennae (Fig. 3A). SSR is a technique used to extracellularly measure the activity of single ORNs within a single sensillum (Berg et al. 1995; Hull et al. 2004). During SSR recordings, the butterfly or moth is restrained in a holder, and a sharp recording electrode is inserted into the sensillum of an antenna. When the sensillum is exposed to odors, such as pheromones (Grant et al. 1989) or plant-related compounds (Schuh et al. 2015; Shields and Hildebrand 2001), the generated action potentials of a single ORN can be measured.

In addition to olfaction, many studies in Lepidoptera have focused on the color vision system, in particular in Papilionoid and Nymphalid butterflies. Here, electroretinography (ERG) provides a classic approach to reveal the spectral sensitivity of photoreceptors in the eye (Cowan and Gries 2009; Eby et al. 2013; Steiner et al. 1987; Swihart 1964, 1972). By inserting an electrode into the retina of a butterfly’s compound eye, the combined response of a population of photoreceptors to a given light stimulus can be measured extracellularly (Fig. 3A). ERG recordings have provided valuable insights into the ecological adaptations of butterfly visual systems and adaptations to different habitats or lifestyles (Chatterjee et al. 2020; Crook et al. 2022; Martín-Gabarrella et al. 2023). However, it is not trivial to isolate the spectral sensitivity of a certain type of photoreceptor from ERG recordings. To achieve this, researchers have performed intracellular single photoreceptor recordings (SPR, Fig. 3A) from the butterfly eye using a sharp glass electrode (Arikawa et al. 1999; Blake et al. 2020; Ilić et al. 2022; Nagloo et al. 2020; Pirih et al. 2018; Satoh et al. 2017). Combined with a visual stimulus that allows the presentation of specific wavelengths, this technique has shown that the eyes of some butterflies, such as *Papilio* (Chen et al. 2016; Wakita et al. 2024), are equipped with up to nine different types of photoreceptors, and that butterflies possess photoreceptors responsible for the detection of polarized light (Belušič et al. 2017; Stalleicken et al. 2006). As the recording electrode can be filled with a tracer, subsequent anatomical identification of the exact photoreceptor type within an ommatidium is possible, allowing a direct comparison of visual systems between different butterfly species (Belušič et al. 2021).

Similarly, many studies have applied intracellular recordings combined with tracer injections to identify and physiologically characterize neurons in the brain (Céchetto et al. 2022; Hansson et al. 1992; O’Carroll et al. 1996). Remarkably, due to low levels of variation between individuals, this method has allowed researchers to even perform recordings from the same neuron in different individuals. In combination with odor stimulation, the neural circuitry of insect olfaction was first described in the sphinx moth, *Manduca sexta* using intracellular recordings (Kanzaki et al. 1989, 1991; King et al. 2000; Matsumoto and Hildebrand 1981; Reisenman et al. 2005, 2011). Neurons likely involved in the motor control of pheromone tracking in *Bombyx mori* (Iwano et al. 2010; Mishima and Kanzaki 1999; Namiki et al. 2018), and the neural mechanisms of dim-light vision in *Deilephila elpenor* (Stöckl et al. 2016, 2017, 2020), were also first described in insects by the means of intracellular recordings. Beyond this, intracellular recordings were paramount to the discovery of neurons involved in long-distance migration in butterflies (Heinze and Reppert 2011; Nguyen et al. 2021, 2022) and moths (Dreyer et al. 2025). Take together, intracellular recordings combined with tracer injections have allowed researchers to set the groundwork for understanding where different sensory modalities, such as olfaction (Chaffiol et al. 2012; Chu et al. 2020; Løfaldli et al. 2012; Namiki et al. 2008), vision (Kinoshita and Stewart 2022), and audition (Pfuhl et al. 2014, 2015; Zhao et al. 2013) are processed in the lepidopteran brain.

However, some research questions require us to observe the activity of a population of neurons in the brain rather than looking at the isolated response of a single cell. To achieve this, extracellular recordings using multi-channel silicon microprobe arrays have been performed on the brains of several lepidopteran species (Lei et al. 2004; Riffell et al. 2009, 2013), for example, to reveal the dynamics of olfactory coding in the antennal lobe of *Manduca sexta* (Christensen et al. 2000). These recordings have the great advantage of being relatively stable, and the capacity for long-term monitoring of neural activity enables them to be combined with other systems, such as gas chromatography, to reveal the processing of odor information in the lepidopteran antennal lobe (Riffell et al. 2009). Similarly, a number of studies have used optical imaging to study the coding in the lepidopteran antennal lobe. By inserting a calcium indicator into antennal lobe neurons and detecting their calcium signal in a moth placed under a fluorescence microscope, the response of the glomeruli, functional units in the antennal lobe, have been analyzed in detail (Bisch-Knaden et al. 2018, 2022; Galizia et al. 2000; Hansson et al. 2003; Ian et al. 2017; Kuebler et al. 2011; Kymre et al. 2021; Meijerink et al. 2003; Skiri et al. 2004). These methods have substantially advanced our understanding of how neural populations and brain regions, such as the lepidopteran antennal lobe, map odor information in space and time.

### Neural recordings in active butterflies and moths

All neurophysiological methods mentioned so far have been performed on immobilized lepidopterans. However, several studies have reported clear evidence that locomotor activity modulates neural coding in the insect brain (Maimon et al. 2010; Weir and Dickinson 2015). To consider such changes, recent studies have successfully developed neural recordings from tethered individuals, such as flying Monarch butterflies, *Danaus plexippus* (Beetz et al. 2022, 2023). In these experiments, butterflies were tethered at the center of a flight simulator and were free to steer with respect to a simulated sun. By simultaneously observing the orientation behavior and the activity of neurons, using extracellular multichannel tetrode recordings (Fig. 3A), different neural cell types of the Monarch sun compass system were described physiologically (Beetz and el Jundi 2023). Although these recordings can be used to reliably obtain neural data from the same brain region in different flying individuals, recordings from the same neurons in different animals cannot be reliably achieved. As such, while tetrode recordings are ideal for investigating how the brain of lepidopterans control behavior under more naturalistic conditions, the inability to perform these recordings from specific neurons represents a major limitation.

### Genetically encoded tools for neural recordings

The growing application of genetic tools in butterflies and moths (Iiams et al. 2019, 2024; Merlin et al. 2013; Wan et al. 2021; Zhang et al. 2017), including studies that investigate neural coding in knockout mutants (Fandino et al. 2019), suggests that genetically modified lepidopterans with labeled cell populations in the brain will become technically feasible soon (Fig. 3B; Section “Genetic manipulation of brains and behavior”). Such an advance would enable lepidopteran neuroethologists to execute similar experiments to those performed in *Drosophila*. Here, using the combined power of virtual reality and sophisticated genetic tools has made it possible to perform neural recordings from genetically labeled neurons in tethered, flying *Drosophila*. This was achieved by mounting head-fixed transgenic GAL4 flies below a fluorescence microscope, with the head capsule opened to permit access to the brain. Labeled neurons could then be targeted and recorded intracellularly using whole-cell patch clamp recordings (Maimon et al. 2010). Using split GAL4 driver lines in flies, in conjunction with UAS-mediated expression of genetically encoded calcium indicators even allows imaging the activity of specific cell populations through optical two-photon calcium imaging in virtual reality systems (e.g. Green et al. 2017; Mussells Pires et al. 2024). Although several studies have already successfully placed flying (Gray et al. 2002) or walking (Yamada et al. 2021) lepidopterans in experimental virtual reality systems, the lack of lines with labeled neurons remains a major drawback when using lepidopterans to study brain function. Lepidopteran researchers who performed calcium imaging in the past have relied on introducing the calcium indicator into the cells through injections into tracts (Kymre et al. 2021), or by allowing a calcium indicator to diffuse into the brain tissue and enter cells (Bisch-Knaden et al. 2022). This restricted the use of optical imaging towards research questions with easily accessible brain regions at the brain surface, such as the antennal lobe.

In summary, a range of established neurophysiological methods have already placed several lepidopteran systems as critical case studies in our understanding of a range of behavioral processes, from sensory perception to goal-oriented behavior. Nevertheless, biases persist in the neural cells and structures that are currently amenable to recordings. Future integration of molecular tools with current technologies will shift some of these biases. This will be enabled by increased data on cell types, the generation of cell type specific regulatory regions, and more advanced genetic tools. Developing genetically encoded lines for neurophysiological studies is especially attractive in lepidopterans given that they are amenable to experiments in both virtual reality systems in the laboratory (Dreyer et al. 2025; Franzke et al. 2020, 2022; Gray et al. 2002; Yamada et al. 2021) and in nature (Dreyer et al. 2018a, b; Merlin et al. 2009; Mouritsen and Frost 2002; Reppert et al. 2004), offering a unique window into the driving evolutionary and ecological forces and their impact on the coding of neural circuits in actively behaving animals.

## Genetic manipulation of brains and behavior

Manipulating the genome to directly test how genes influence neural function and natural behavior, or to visualize neural circuits and their activation, is paramount to the field of functional neurogenetics, and has been critical to the success of bridging genetics and behavior. Neuro-geneticists working in model organisms now have access to a plethora of tools, including a large collection of transgenic lines in *Drosophila* flies (e.g. split-Gal4 drivers lines), that allow researchers to visualize or activate single neurons (Meissner et al. 2025). For example, in a technical tour de force, Ding et al. used a neurogenetic approach to identify a pair of neurons that control courtship song in two *Drosophila* species that produce divergent song types (Ding et al. 2019). Inhibiting these neurons caused almost complete elimination of mating songs in both species, while optogenetic activation of these neurons in freely behaving flies triggered song production, demonstrating a remarkable ability of these neurons to drive specific behaviors.

While there is no other insect that is remotely close to this level of manipulability, there is widespread interest in developing neurogenetics in a range of insects, including *Tribolium* (Farnworth et al. 2020; Rethemeier et al. 2025), Hymenoptera (Carcaud et al. 2023; Hart et al. 2023), mosquitoes (Weiss and McBride 2024), and Lepidoptera (Bisch-Knaden et al. 2022; Kymre et al. 2021). In conjunction with the extensive history of lepidopteran neuroethology, and a huge amount of genetic data, butterflies and moths offer great potential to study how olfactory and visual systems guide a range of behaviors. Currently, experimental genetic modifications mainly rely on two techniques: transgenesis based on the random insertion of recombinant DNA by transposases, and genome editing based on the use of programmable nucleases such as CRISPR. Below, we summarize advances and challenges associated with the use of transgenesis and genome editing, and propose future avenues of optimization for comparative lepidopteran neurogenetics.

### Transposase-mediated transgenesis in silkworm neurogenetics

The silkmoth, *Bombyx mori*, has been a flagship model for lepidopteran functional genomics, and benefited from the development of transgenesis protocols more than 25 years ago (Tamura et al. 2000) mainly using the piggyBac transposase system. Transposase-mediated approaches generate random insertions of genetic content that label randomly captured genetic contexts. While many transgenic lines have been developed in this system, including UAS and GAL4 lines that allow combinatorial assays for the study of gene expression and function in specific tissues, few studies have used these technologies to directly study *Bombyx* neurons, nervous system or behavior (Kiya et al. 2014). As a notable exception, neurogenetics tools based on transgenesis have shed important insights into the sensory basis of pheromone olfaction (Sakurai et al. 2011; Fujiwara et al. 2014; Hara et al. 2017). Here, Sakurai et al. cloned the promoter of the olfactory receptor gene involved in pheromone reception (*BmOR1*) to drive the expression of the *OR1* ortholog from a distant species, the diamondback moth, *Plutella xylostella* (Sakurai et al. 2011). Remarkably, this experiment elicited responsiveness of the transgenic *B. mori* males to *P. xylostella* pheromones and live females, suggesting that the neuronal circuitry downstream of the olfactory receptor can interpret novel pheromone inputs. This finding implies that species-specific mate recognition in moths can be modified by altering a single receptor, highlighting the key role of olfactory tuning in species divergence.

To further investigate the neuronal bases of pheromone reception, Fujiwara et al. (2014) generated transgenic *Bombyx* expressing *GCaMP2*, a genetically encoded calcium indicator, in the *BmOR1*-expressing olfactory receptor neurons (ORNs) that are responsive to Bombykol, the female-calling pheromone. Calcium responses to bombykol pulses increased in a concentration-dependent manner, and comparing the responses of ORNs and projection neurons (PNs) in the antennal lobe revealed that the transformation of odorant concentration coding occurs downstream of the ORN-PN synapses, likely due to inhibitory feedback. Later, Hara et al. refined the GAL4/UAS system to visualize neuronal tracts, measure neural activity using calcium imaging, and perform targeted neuron inhibition (Hara et al. 2017). Using increased copies of GAL4 binding sites and an N-myristoylation signal (myrGFP), bright labelling of axonal tracts was obtained, which showed that *BmOR1*-expressing cells converge their axons onto a single glomerulus, called the ‘toroid’. Finally, Hara et al. drove the expression of Tetanus Toxin Light Chain (TeTxLC) to block synaptic transmission in the Bombykol-responsive ORNs. This targeted blocking successfully inhibited male courtship behavior, demonstrating the effectiveness of genetically targeted toxins for perturbation analyses of neural circuits involved in pheromone detection.

### CRISPR approaches for targeted gene knock-outs and knock-ins

Alongside transposase-based approaches, programmable nucleases used in CRISPR and TALEN genome editing have also been successfully applied to lepidopteran species (Ahmed et al. 2025). These allow the generation of DNA double strand-break at targeted sites (encoded in a ‘guide’ molecule). These breaks are spontaneously repaired by the Non-Homologous End Joining (NHEJ) pathway, which is error prone, generating frameshift mutations within a coding gene which results in protein null mutants, or somatic “crispants” (a term highlighting the mosaic nature of injected individuals at the G_0_ generation). This technique has become an essential testing tool to assess the function of genes in olfaction, vision, and behavior. Sensory proteins including olfactory receptors, including the odorant co-receptor (Orco), and photoreceptors have been select targets of knock-out experiments, which confirmed their necessary roles to a variety of behaviors (Koutroumpa et al. 2016; Revadi et al. 2021; Chang et al. 2017; Fandino et al. 2019; Chen et al. 2025; Liu et al. 2023; Wang et al. 2024; Cao et al. 2023; Tang et al. 2024).

CRISPR knock-outs have also been used to assess behaviors beyond the peripheral sensory systems. The remarkable navigational capabilities of *Danaus plexippus* have been the focus of molecular investigations using TALEN and CRISPR deletion experiments. For example, loss-of-function mutants for circadian clock genes like *Clock*, *Bmal1*, and *Cry2* abolished photoperiodic responses in reproductive output, demonstrating the necessity of these genes for sensing the seasonal changes that trigger shifts in monarch physiology and behavior (Zhang et al. 2017, 2023; Iiams et al. 2019). Similarly, CRISPR mutants for *ninaB1*, encoding a rate-limiting enzyme in the vitamin A pathway, revealed a role in photoperiod responsiveness independently of visual function (Iiams et al. 2019). While the vertebrate-like cryptochrome Cry2 regulates circadian transcription, it appears dependable for magnetoreception in monarchs. Instead, its insect-specific Cry1 paralogue is required for Monarchs to detect changes in magnetic field orientations that are on par with Earth magnetic intensities (Iiams et al. 2019; Merlin 2023).

Alongside *Danaus*, *Heliconius* butterflies have played a leading role in applying CRISPR to natural butterfly behavior. *Heliconius* show complex mating behaviors that can be quantified in the lab, and genetic studies have identified loci that underlie the preference of males for certain wing color patterns during courtship behavior (Rossi et al. 2024; VanKuren et al. 2025). Rossi et al. found that two *Heliconius* butterfly species (*melpomene* and *timareta*) evolved similar preferences for red wing patterns through adaptive introgression of a major-effect locus that includes the *regucalcin1* gene. CRISPR-Cas9 knockouts of *regucalcin1* disrupted male courtship, confirming its role in mating behavior (Rossi et al. 2024). In addition, differential expression between species suggested that its *cis*-regulation is associated with visual preference. Another *Heliconius* locus under investigation drives preference for yellow or white patterns, and appears to function in the peripheral sensory system (VanKuren et al. 2025). These studies of behavioral evolution in *Heliconius* open new avenues of research on the neuronal basis of sensory processing in these large-brained butterflies (Couto et al. 2023; Farnworth et al. 2024).

**Fig. 4:**
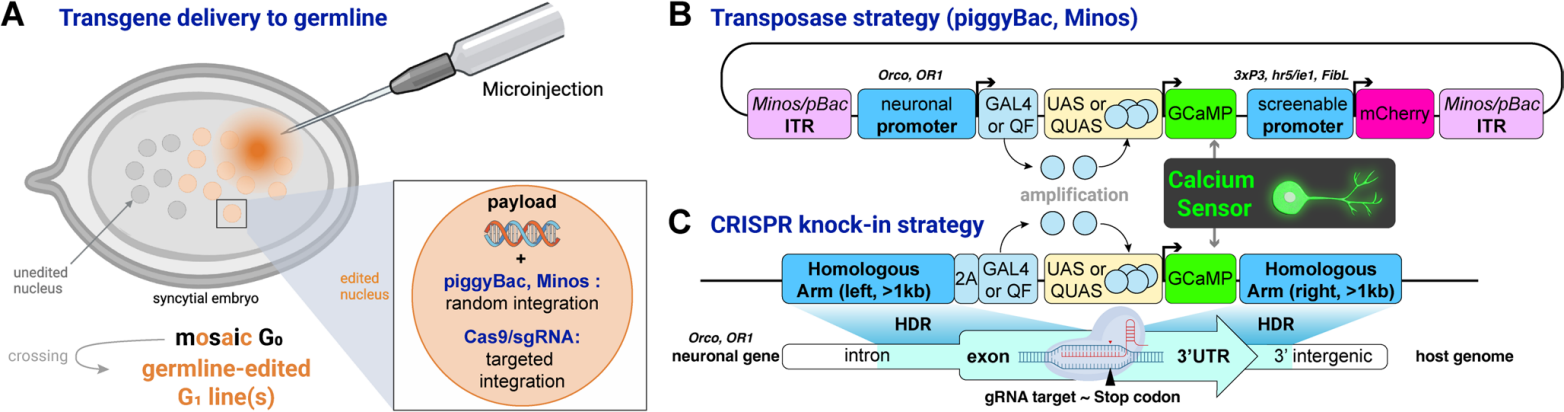
Strategies for genome integration, expression and amplification of calcium sensors or other neurogenetic tools in neuronal populations. **(A)** Delivery of transgenes to the germline requires the injection of syncytial embryos collected shortly after fertilization. Injected individuals (G_0_ generation) form mosaics and requires further crossing for stabilization into the germline. **(B)** Transposase-based strategy for the integration of GCaMP under the activation of a neuron-specific promoter, similar to a strategy previously used in ants (Hart et al. 2023). Internal terminal repeats (ITRs) are used for payload recognition and integration by the corresponding transposase. GCaMP is a genetically encoded calcium indicator, consisting of a fusion of green fluorescent protein (GFP), calmodulin (CaM), and M13. A promoter-driven fluorescent protein is used as a transgenesis marker. We recommend a monomeric red-fluorescent protein such as mCherry due to inconsistent results with DsRed in *Plodia* moths. **(C)** CRISPR knock-in strategy for the integration of GCaMP in frame with a neuron-expressed protein, using Homology-Directed Repair (HDR). The 2 A ribosome-skipping sequence can assist in maintaining native gene function while producing ectopic protein. This strategy has been used in mosquitoes (Zhao et al. 2022)

Undoubtedly, CRISPR knock-outs will continue to provide insights into the genetic basis of species-specific behavior in systems like *Danaus* and *Heliconius*. However, when coupled to repair templates, CRISPR edits should also allow the insertion of transgenes that function as neurogenetic tools. As an example, in the mosquito *Aedes aegypti* (Zhao et al. 2022), CRISPR was used to knock-in the *GCaMP6* calcium reporter insert at the stop codon of the *Orco* gene. This strategy was similar to the aforementioned transgene carrying an *Orco* gene promoter in clonal raider ants (Hart et al. 2023), as both studies leverage the regulation of *Orco* in specific olfactory circuits and used the Q-system, an alternative to the GAL4/UAS system (see Section “Technical considerations for neurogenetics in other lepidopterans”), to amplify the expression of *GCaMP6* and enable its detection. Of note, in the *A. aegypti* CRISPR approach, the native transcription of *Orco* was captured to produce a polycistronic QF transcription factor, which in turns activated *GCaMP6* under the control of a *QUAS* (QF-responsive) promoter (Fig. 4C). To our knowledge, the ability to deliver a payload of several hundred base pairs using CRISPR knock-in strategies is still limited in Lepidoptera, because of the reduced chance of successful integration of larger constructs. The development of techniques using transgenic lines expressing the Cas9 CRISPR enzyme in the germline (Zhang et al. 2018; Xu et al. 2022), taking advantage of alternative repair pathways such as NHEJ insertions (Rethemeier et al. 2025; Matsuoka et al. 2025) and Microhomology-mediated end joining (Nakade et al. 2014; Sakuma et al. 2016), or using single-stranded DNA donor templates (Connahs et al. 2022; Heryanto et al. 2022a, b; Tsubota et al. 2025), require further optimization in Lepidoptera before CRISPR can replace classic transgenesis.

### Technical considerations for neurogenetics in other lepidopterans

Studies of *Bombyx* pheromone reception provide proof-of-concept strategies for studying butterfly and moth neuroethology, using genetic labeling of neuronal circuitry, calcium imaging of small neuronal populations, and the targeted expression of ectopic proteins including inhibitory toxins. We foresee five immediate challenges that can be overcome in the near future while developing genetic tools. As we believe the technical detail will be beneficial for the community, we include specific information that may not be immediately accessible to the general reader.

First, with transgenic approaches, it is necessary to develop strategies to identify individuals that carry the introduced transgene. The *3xP3* marker, used to activate a fluorescent protein such as EGFP or mCherry, provides a convenient way to screen transformants (Thomas et al. 2002). Regardless of the tissue opacity in a given species, it universally provides bright labelling of the stemmata, which can be screened in late embryos through the chorion, or in live larvae. However, *3xP3*-driven fluorescence also labels the pupal and adult retina, and glial cells of the nervous system, which can interfere with further experiments of nervous tissues. To circumvent that, the *hr5/ie1* and *Opie2* viral promoters have also been widely used as transgenesis markers in Lepidoptera (Xu and O’Brochta 2015; Martins et al. 2012) and more recently, silkworm neurogenetics studies have used marker that leverage a *Fibroin Light chain* (*FibL*) promoter to drive fluorescence in the silk glands (Fujiwara et al. 2014).

Second, for most species, generating and maintaining stable transgenic lines will be unfeasible. The *Bombyx* research community has primarily relied on the GAL4/UAS system. This binary system includes two components, a tissue-specific driver line expressing GAL4, and a responding line that uses UAS (GAL4-responsive) promoters to amplify a reporter (Li et al. 2014). This method provides greater sensitivity and detectability in assays such as calcium imaging. While experimentally powerful, a binary system involves the long-term maintenance of transgenic lines, and this may not be possible in lepidopteran organisms that are sensitive to inbreeding depression or disease, or that require considerable human intervention for rearing and husbandry. Even for species for which transgenic lines have been maintained over several generations, it may be unrealistic to maintain more than a handful of lines over several years. To circumvent this challenge, it should be possible to use transgenic constructs that combine a transgenesis marker and a single transgene of interest instead of a modular system such as UAS/GAL4 systems. As an example, Hart et al. generated a transgenic line of the clonal raider ant that allowed calcium imaging of the olfactory response to exposure to alarm pheromones (Hart et al. 2023; Schulte et al. 2014; Yan et al. 2017). To allow good sensitivity of calcium signals, GCaMP6 was driven by the *Orco* co-olfactory receptor promoter and amplified via the Q-system encoded on the same plasmid. The *Orco* promoter drives the yeast transcription factor QF, which in turn activates the QUAS response element driving *GCaMP6* at the same transgene (as in Fig. 4B). The Q-system is similar in concept to GAL4-UAS but less prone to silencing (Riabinina et al. 2015), and has been preferred in recent years in the field of mosquito neurogenetics (Giraldo et al. 2024; Zhao et al. 2021). Overall, this strategy is sound for neurogeneticists interested in developing calcium imaging in lepidopteran insects.

Third, *piggyBac* transposons, used for random insertion mediated transgenics, actually derive from a transposon that was originally isolated from a lepidopteran, the Cabbage Looper, (*Trichoplusia ni*; Fraser et al. 1985). This family of transposases is encoded in many lepidopteran genomes and endogenously active, implying it may be able to remobilize transgenes with *piggyBac* terminal repeats. If this is the case, remobilized transgenes may cause genomic instability and sterility, or be confined to silenced regions of the genome, inhibiting desired experimental effects. While the *Hyperactive piggyBac*, a more active, bio-engineered version of the transposase shows high rates of transformation in Lepidoptera (Chen and Palli 2021; Heryanto et al. 2022b). Recent work with the *Minos* transposase has also shown promise with efficient transformation rates (Uchino et al. 2007; Shodja et al. 2025) and may represent a safer alternative given its dipteran origin (Franz and Savakis 1991).

Fourth, and counterintuitively, CRISPR cutting is sometimes too efficient, and decreasing the efficiency of cutting might favor the frequency of knock-ins over NHEJ knock-outs. During knock-in experiments, a donor sequence is provided as a repair template, usually on a small circular piece of DNA called a plasmid. For knock-ins to occur, this template must be in the nucleus at the point in which the double-strand break is made by the CRISPR nuclease. If a CRISPR nuclease is introduced into a cell as a protein-sgRNA duplex (i.e. the guide sequence which localizes to the target site and nuclease are physically linked), it may arrive too fast at its target site in the genome. If it arrives and cuts the DNA at the target site before any repair donor DNA molecule is present in the nucleus, a NHEJ repair will take place, likely introducing errors that will make this site unavailable for further editing. To circumvent this, it may be helpful to encode the transcription of the guide RNA on the same plasmid that carries the donor repair template, ensuring both are present in the nuclei that have incorporated the exogenous DNA molecule. This strategy has been prevalent across model organisms, and has been more recently repackaged in *Drosophila*, resulting in homology-directed repair with higher efficiency than previously observed in this system (Stern et al. 2023). Specific promoters, the U6 promoters, have been widely used for gRNA transcription in Lepidoptera (Huang et al. 2017; Chen and Palli 2023; Zeng et al. 2016), and can be flanked by tRNAs for improved processing (Port and Bullock 2016). While it is too early to predict whether transposon-based or CRISPR-based insertions of long transgenes will prevail in emerging model systems for neurogenetics, there is undoubtedly room for enriching the toolkit that will enable deeply mechanistic studies of behavior in Lepidoptera.

Finally, both random integration and targeted editing techniques discussed above rely on microinjecting freshly fertilized embryos (Fig. 4). Unless microinjection can be performed within minutes after egg laying, only a subset of the dividing nuclei present in the embryonic syncytium tend to undergo modification. Practically speaking, this means that G_0_ individuals (i.e. the injected generation) carry genetic modifications in a ‘mosaic’ state, meaning that only a fraction of the soma and germline potentially integrated a genetic change. As such, for many studies of behavior, a secondary challenge of neurogenetics is to provide individuals that are homozygous for the modified allele. If the G_0_ offspring are healthy and fertile, edits are passed via the germline into a G_1_ generation which can then be called “germline transformants”. In-crossing G_0_ individuals (i.e. G_0_ x G_0_ matings) can generate compound heterozygotes that carry different versions of the intended modifications, with different mutations at CRISPR repair sites, or different transgene insertion sites. Thus, proper genotyping is necessary to control for this heterogeneity in subsequent generations, and further out-crossing can assist in reducing the number of alleles if preferable. Alternatively, G_0_ individuals can be out-crossed to a non-injected stock, and will generate some G_1_ individuals that will carry a single allele of the intended modifications in a heterozygous state. Further G_1_ sib-matings can then lead to a mix of heterozygous and homozygous carriers if needed. While closer to the standards of model organisms, this strategy is more amenable to lepidopteran systems in which controlled crossings are practical.

In summary, among non-model organisms, experimental manipulation of genes for tool development or hypothesis testing has a strong history in Lepidoptera, with some notable success stories in coupling functional genetics with evolutionary case studies (Rossi et al. 2024; VanKuren et al. 2025). Although technical challenges remain, and some aspects of lepidopteran biology may require modifications to methods developed in model organisms, ongoing advances in transgenesis and genome editing promise effective visualization and manipulation of neural circuits and behavior.

##  Conclusions and prospects

In this review we aimed to reflect on established and emerging methods in understanding lepidopteran brains and behavior, and prospects for their future application. We emphasize that a core strength of utilizing butterflies and moths as study systems is their behavioral diversity, and the foundation provided by the phylogenetic and ecological literature to develop research programs based in the natural challenges Lepidoptera face, and how these vary across species, or within species, between sexes or seasons. While the methods described above can be used to further advance established work in butterflies and moths, we also see scope for taking advantage of the many understudied behavioral innovations in Lepidoptera. To illustrate how the approaches discussed above can be combined, we provide a potential program for developing new butterfly and moth case studies:

- **Identify your biological target**: identifying a behavior to explore is a critical first step in any neuroethological study. Here, two main approaches have proven successful in the past: (1) identifying marked novelty or extreme phenotypes, where effect sizes of variation in the underlying neural or molecular traits are expected to be pronounced and easier to identify when compared across species (e.g. Beetz et al. 2022; Couto et al. 2023); and (2) identify behavioral variation between closely related species, or polymorphisms within species, where quantitative genetics may be employed to identify candidate mechanisms, or where a background of general conservation may allow divergence in a more limited number of traits to be identified (e.g. Montgomery et al. 2021; Rossi et al. 2024; VanKuren et al. 2025). Careful consideration must also be given to the contexts in which a species will display a given behavior, if they are not amenable to controlled rearing or do not display natural behaviors in relatively controlled contexts, they are unlikely to be productive long-term study systems for neuroethology, but could of course form the basis of productive field-based neuroecological research.
- **Assess the ecological and phylogenetic context**: The comparative approach is one of our most productive tools, comparing variation between populations or species not only identifies variation, but can provide evidence of adaptation. But it is most appropriate when embedded in a phylogenetic framework in the context of sound understanding of the species’ ecology and behavior (Pagel 1999; Revell and Harmon 2022). Understanding the distribution of traits across related species, or larger samplings of the lepidopteran phylogeny, and testing for co-evolution between neural and behavioral variation (e.g. Snell-Rood et al. 2020; Couto et al. 2023; Young et al. 2023), or between behavior and ecological variation can provide grounding insights in themselves, but also direct future functional studies. Indeed, often macroevolutionary patterns in more crude metrics like volumes of brain structures provide indications of underlying cellular change, greatly narrowing down where in the sensory or nervous systems we should focus our studies.
- **Developing genomic resources**: Any neuroethological system must be experimentally tractable, and although many questions can be answered without molecular resources, as discussed above they can greatly extend the scope for functional insight. A well assembled, contiguous genome, with protein coding loci (including UTRs) and regulatory elements annotated using RNA-seq and ATAC/Chip-seq data provides the basis of downstream analyses. These can include phylogenomic approaches to assess conservation/rapid evolution of genomic regions, or gene-phenotype co-evolution across phylogenomic datasets (e.g. Cicconardi et al. 2023), but are also an essential basis for single-cell approaches to cataloguing cell types and the spatial distribution of those cells (see Section “Molecular tools to study neural diversity in Lepidoptera”). In turn, cell type markers and/or candidate gene regions of interest provide the basis of a more advanced package of tools.
- **Assaying neural activity**: Understanding the activity of neurons during behavior is essential for dissecting the relevant neural pathways involved. Developing brain atlases and making comparisons across populations/species may reveal target sights for analyses, but the largely conserved architecture of insect brains may also mean some systems can utilize insights from established model species, including the *Drosophila* connectome. While molecular approaches (see Section “Dynamic gene regulation of neural cells”) and established methods such as tetrode recordings are still productive tools, molecular methods including cell-type specific Calcium indicators combined with advanced microscopy may allow more flexible and precise recordings in the future (see Section “Advances in neurophysiological recordings in free moving Lepidoptera”). Combined with the development of tethered flight arenas, and virtual reality, there is great scope for future advancements in recording neural responses during natural, behavioral expression.
- **Identifying candidate genetic mechanisms**: Linking brain and behavior is a major challenge. The tools of comparative biology offer productive approaches to testing associations predicted by our adaptive or mechanistic hypotheses, but are limited in their potential to demonstrate causation. Here, disruption or manipulation of neural processes provides the most direct route to causative effects on behavior. But we should not be interested just in how to *break* a system, but in how evolution has *changed* it. As such, identifying candidate loci involved in the evolution of behavior and the neural systems that have evolved to support that change, is critical. The approach taken may depend on the phylogenetic distribution of a phenotype’s variation, but phylogenomic and transcriptomic data provide an accessible path to identifying genomic loci with deviant patterns of molecular evolution, or deviant patterns of gene regulation (e.g. Cicconardi et al. 2023), while quantitative methods have been used to successfully map loci affecting variation in behavioral traits among close relatives (Rossi et al. 2024; VanKuren et al. 2025). Recent improvements in our ability to identify regulatory elements through ATAC/Chip-seq, and through analysis of aligned genomes, is critical as these regulatory elements are likely less pleiotropic, so are more likely to be involved in evolutionary change and more likely to show precise phenotypes when manipulated.
- **Transgenic tests of mechanistic hypotheses**: Once a locus is identified, a number of tools will be deployable in the future (see Section “Advances in neurophysiological recordings in free moving Lepidoptera”). CRISPR can provide knock-outs at acceptable, but low, rates to explore loss of function traits. New transgenic approaches using transposases may allow insertion of alternative alleles (e.g. swapping regulatory sequences between species to observe reciprocal changes in development). They can also be used to reveal where in the nervous system a gene is expressed, by linking the regulatory sequence of interest to fluorescent reporter constructs to, or to analyze neural activity by linking a regulatory sequence to a calcium reporter. These methods are in their infancy in most lepidopteran systems, but they mark an exciting new endeavor in tool development that can be applied across a range of experimental contexts.

To support the community in the establishment of these methods in new laboratories and contexts, we have established an open-source library of protocols relevant for neuroethology in butterflies and moths, with an initial set of resources covering dissection, immunohistochemistry, transgenesis and tetrode recordings (DOI: 10.17605/OSF.IO/JDM62). By adding to this primer over coming years, we hope the community will collectively generate a rich collection of approaches and a place for the exchange of expertise, and thus support each other to further develop this multidisciplinary field.

## Supplementary Information

Below is the link to the electronic supplementary material.

Supplementary Material 1

## Data Availability

No datasets were generated or analysed during the current study.
