# Supplementary material for: Emerging tools to advance neuroethology in butterflies and moths
Source: J Comp Physiol A Neuroethol Sens Neural Behav Physiol. 2025 Dec 10;212(2):433–57. doi: 10.1007/s00359-025-01785-y (PMC13086684; doi:10.1007/s00359-025-01785-y)
Supplement: Supplementary file 1 — Supplementary Material 1 [file 359_2025_1785_MOESM1_ESM.docx]

**Table 1: A comparison of single cell/nuclei RNA sequencing methods**

| Approach | Platform | Methodology | Advantages | Limitations |
| --- | --- | --- | --- | --- |
| **Plate based methods** | Smart-seq2  (Picelli et al 2014)  So-Smart-seq  (Wei & Lee 2025) | Full-length transcript sequencing from single cells. | High sensitivity and accuracy, detailed transcript information (strand specificity and/or multiple classes of RNA molecules). | Prone to batch effects, low throughput, labour intensive. |
| **Microfluidic methods** | 10x Genomics Chromium  (see Danielski 2022 for review) | Encapsulates single cells with barcoded beads in oil droplets, enabling high-throughput processing of thousands of cells. | Scalability and efficiency in processing large numbers of cells. Suitable for small labs. | Low capture efficiency, increased presence of doublets and multiplets, technical complexity due to the fabrication and operation of microfluidic devices requiring specialized equipment and expertise |
|  | Drop-seq  (Bageritz et al 2019) | Captures single cells with barcoded beads, facilitating cost-effective transcriptome profiling. |  |  |
|  | Fluidigm C1 | Employs microfluidic chips to capture and process individual cells in separate chambers, suitable for detailed analyses, lower throughput compared to droplet-based systems. |  |  |
| **Combinatorial indexing methods (split-pool)** | SPLiT-seq  (Kuijpers et al 2024); commercially available from Parse Biosciences | Applies successive rounds of barcoding in bulk cell populations, enabling the profiling of thousands to millions of cells. | Ultra-high throughput capability, reduced batch effects through simultaneous processing of vast numbers of cells, no need for physical isolation of individual cells, reduces reliance on expensive microfluidic devices, lower per unit cost. | Complexity in the library preparation, the multi-step barcoding process can be technically challenging and may require extensive optimization; barcode misassignment, due to errors in barcode assignment, which can lead to incorrect cell identification, affecting data quality. |
|  | sci-RNA-seq and FIPRESCI  (Li et al 2023) | Combine droplet microfluidics with combinatorial indexing to enhance throughput and reduce costs. |  |  |

**Table 2: A comparison of spatial transcriptomics methods**

| Approach | Platform | Methodology | Advantages | Limitations |
| --- | --- | --- | --- | --- |
| **Array-based platforms** | 10X Genomics Visium | mRNAs are captured from tissue sections using spatially barcoded arrays (analogous to pixels) at a resolution of ~55 μm (Ståhl et al 2016). | Suitable for large tissues with relative homogeneity of cell types. | Limited resolution and spatial accuracy of the detected mRNA. |
|  | 10X Genomics Visium HD | As above. | Dramatically increases the resolution to 2 μm by miniaturizing the capture grid (Oliveira et al 2024). |  |
| **Bead-based platforms** | Slide-seqV2 (Curio Seeker)  High-Definition Spatial Transcriptomics (HDST) | Densely barcoded bead arrays, termed ‘pucks’, are fabricated by split-pool phosphoramidite synthesis and indexed up front using a sequencing-by-ligation strategy. Randomly arrayed or deterministically placed beads, enables finer resolution. | Improved spatial resolution (~10 μm for Slide-seq V2; subcellular resolution for HDST) (Stickels et al 2021).  Require only the cryo-preservation of samples. | Cryostat needed. |
| **Polony-/Nanoball- based platforms** | Stereo-seq | DNA nanoballs or polonies (DNBs) are small, circular DNA structures, typically 220nm in diameter, each with a unique barcode sequence, which acts as a spatial identifier. DNBs are arranged in a patterned array on a chip, with each DNB occupying a specific location. DNB barcodes are sequenced *in situ* conserving spatial information within a tissue (Chen et al 2022). | Improved spatial resolution; distances between spot centers are smaller than 10 μm and spots in them are binned into 10 μm-sized spots for visualization.  Require only the cryo-preservation of samples. | Specialist equipment needed.  Deep sequencing and optimisation of protocols required. |
| **Microfluidic-based platforms** | DBiT-seq | RNA is captured and barcodes are hybridised *in situ* using microfluidic devices using a microfluidic chip containing parallel microchannels (Liu et al 2020). |  |  |

**References:**

Bageritz J, Willnow P, Valentini E, Leible S, Boutros M, Teleman AA (2019). Gene expression atlas of a developing tissue by single cell expression correlation analysis. Nat Methods (8):750-6.

Chen A, Liao S, Cheng M, Ma K, et al. (2022). Spatiotemporal transcriptomic atlas of mouse organogenesis using DNA nanoball-patterned arrays. Cell (10):1777-92.

Danielski K (2022). Guidance on processing the 10x genomics single cell gene expression assay. InS ingle Cell Transcriptomics: Methods and Protocols (pp. 1-28). New York, NY: Springer US.

Kim J, Marignani PA (2022) Single-Cell RNA Sequencing Analysis Using Fluidigm C1 Platform for Characterization of Heterogeneous Transcriptomes. In Cancer Cell Biology: Methods and Protocols (pp. 261-278). New York, NY: Springer US.

Kuijpers L, Hornung B, van den Hout-van Vroonhoven MC, van IJcken WF, Grosveld F, Mulugeta E (2024). Split Pool Ligation-based Single-cell Transcriptome sequencing (SPLiT-seq) data processing pipeline comparison. BMC Genom 25(1):361.

Li Y, Huang Z, Zhang Z, Wang Q, Li F, Wang S, Ji X, Shu S, Fang X, Jiang L (2023). FIPRESCI: droplet microfluidics based combinatorial indexing for massive-scale 5′-end single-cell RNA sequencing. Genom Biol. 24(1):70.

Liu Y, Yang M, Deng Y, Su G, Enninful A, Guo CC, Tebaldi T, Zhang D, Kim D, Bai Z, Norris E, Pan A, Li J, Xiao Y, Halene S, Fan R (2020). High-spatial-resolution multi-omics sequencing via deterministic barcoding in tissue. Cell 183(6):1665-81.

Picelli S, Faridani OR, Björklund ÅK, Winberg G, Sagasser S, Sandberg R (2014) Full-length RNA-seq from single cells using Smart-seq2. Nat Protoc 9(1):171-81.

Ståhl PL, Salmén F, Vickovic S, Lundmark A, Navarro JF, Magnusson J, Giacomello S, Asp M, Westholm JO, Huss M, Mollbrink A, Linnarssno S, Codeluppi S, Borg Å, Pontén F, Costea PI, Sahlén P, Mulder J, Bergman O, Lundeberg J, Frisén J (2016) Visualization and analysis of gene expression in tissue sections by spatial transcriptomics. Science 353(6294):78-82.

Stickels RR, Murray E, Kumar P, Li J, Marshall JL, Di Bella DJ, Arlotta P, Macosko EZ, Chen F (2021) Highly sensitive spatial transcriptomics at near-cellular resolution with Slide-seqV2. Nat Biotechnol 39(3):313-9.

**Supplementary Protocols available at DOI:10.17605/OSF.IO/JDM62**

1. Preparation, dissection and fixation of lepidopteran brains, including a table of antibodies that are cross-reactive in Lepidoptera (DOI: 10.17605/OSF.IO/AC3PJ)
2. Dissection and immunostaining of *Plodia interpunctella* adult brains (DOI: 10.17605/OSF.IO/AC3PJ)
3. Immunostaining, imaging and analysis of Lepidoptera brains (DOI: 10.17605/OSF.IO/VTUZ9)
4. Differential tetrode recording in tethered flying butterflies (DOI: 10.17605/OSF.IO/VE7A9)
5. A portable setup to study butterfly eyeshine (DOI 10.17605/OSF.IO/PH9J6)
6. Lepidoptera eye cuticle dissection and mounting (DOI: 10.17605/OSF.IO/EWBS9)
7. ImageJ/Fiji analysis of eye cuticle, tibia and abdomen measurements of lepidoptera (DOI 10.17605/OSF.IO/FM8VP)
